# nSARS-Cov-2, pulmonary edema and thrombosis: possible molecular insights using miRNA-gene circuits in regulatory networks

**DOI:** 10.1186/s41544-020-00057-y

**Published:** 2020-10-30

**Authors:** P. Khurana, A. Gupta, R. Sugadev, Y. K. Sharma, R. Varshney, L. Ganju, B. Kumar

**Affiliations:** Defence Institute of Physiology and Allied Sciences, Defence R&D Organization, Lucknow Road, Timarpur, New Delhi, India

**Keywords:** nSARS-CoV-2, miRNA coregulatory networks, Pulmonary edema, Thrombosis, Feed forward loops

## Abstract

**Background:**

Given the worldwide spread of the novel Severe Acute Respiratory Syndrome Coronavirus 2 (nSARS-CoV-2) infection pandemic situation, research to repurpose drugs, identify novel drug targets, vaccine candidates have created a new race to curb the disease. While the molecular signature of nSARS-CoV-2 is still under investigation, growing literature shows similarity among nSARS-CoV-2, pulmonary edema, and thromboembolic disorders due to common symptomatic features. A network medicine approach is used to to explore the molecular complexity of the disease and to uncover common molecular trajectories of edema and thrombosis with nSARS-CoV-2.

**Results and conclusion:**

A comprehensive nSARS-CoV-2 responsive miRNA: Transcription Factor (TF): gene co-regulatory network was built using host-responsive miRNAs and it’s associated tripartite, Feed-Forward Loops (FFLs) regulatory circuits were identified. These regulatory circuits regulate signaling pathways like virus endocytosis, viral replication, inflammatory response, pulmonary vascularization, cell cycle control, virus spike protein stabilization, antigen presentation, etc. A unique miRNA-gene regulatory circuit containing a consortium of four hub FFL motifs is proposed to regulate the virus-endocytosis and antigen-presentation signaling pathways. These regulatory circuits also suggest potential correlations/similarity in the molecular mechanisms during nSARS-CoV-2 infection, pulmonary diseases and thromboembolic disorders and thus could pave way for repurposing of drugs. Some important miRNAs and genes have also been proposed as potential candidate markers. A detailed molecular snapshot of TGF signaling as the common pathway, that could play an important role in controlling common pathophysiologies among diseases, is also put forth.

**Supplementary information:**

**Supplementary information** accompanies this paper at 10.1186/s41544-020-00057-y.

## Introduction

Coronaviruses are retroviruses that are sub-categorized as positive sense single stranded RNA viruses [1]. They belong to Order: Nidovirales; Suborder: Coronavirineae; Family: Coronaviridae; Subfamily: Orthocoronavirinae; Genus: Betacoronavirus; Subgenus: Sarbecovirus; Species: Severe acute respiratory syndrome coronavirus 2. Their genomes size is of ~ 30 kb that includes a 5′cap structure and a 3′polyA tail [2]. The different coronaviruses can be further classified into four types: α, β, γ, and δ based on specific genomic and protein regions [3]. Novel Severe Acute Respiratory Syndrome Coronavirus 2 (nSARS-CoV-2) belongs to the β subtype of the coronavirus, which has 45–90% genetic similarity with the other β subtype CoVs such as Severe Acute Respiratory Syndrome-Coronavirus (SARS-CoV) and Middle East Respiratory Syndrome coronavirus (MERS-CoV) [4]. The transcriptomic analysis of nSARS-COV-2 shows that some of the important proteins that are required for virus attachment, viral replication, and pathogenesis are almost consistent in coronaviruses with minor variations. These proteins are spike (S) glycoprotein, matrix (M) protein, small envelope (E) protein, and nucleocapsid (N) protein. Spike glycoprotein (S) functions to bind the virus with the host cell receptor and mediate membrane fusion and virus entry [5]. S protein contains two subunits, S1 and S2, each is about 180 kDa [6]. Recent reports suggested that S protein of nSARS-CoV-2 has a strong interaction with human angiotensin-converting enzyme 2 (ACE2), especially that are expressed on lung alveolar epithelial cells [7]. The M (membrane) protein (also known as E1 membrane glycoprotein or matrix protein) functions together with the S (spike) and the E (envelope) proteins. This protein is the crucial components of viral assembly and morphogenesis, involved in regulation of replication and packing the genomic RNA into viral particles [8]. Another viral protein called small envelope (E) protein having just 76 amino acids, is a membrane component of coronaviruses. This protein plays a significant role in coronavirus life cycle. Another important protein is nucleocapsid (N) protein, which is a structural protein that forms complexes with genomic RNA [9]. The primary function of N protein is to interact with the viral membrane protein and enhance the efficiency of virus transcription and its assembly.

Recent findings have suggested that nSARS-CoV-2 can control the expression of host regulatory molecules such as miRNAs and Transcription Factors (TFs) [10, 11]. Both of these regulatory biomolecules regulate the gene expression at the transcriptional and post-transcriptional level [12]. Gene regulation is viewed as a complex process where regulatory elements and their targets form highly complex network interactions. Construction and analysis of miRNA:TF: gene co-regulatory networks have been successfully implemented in many complex disease models like myocardial infarct, cancer, hypoxia, etc. to analyze multidimensional interactions to identify mechanistic insights and potential targets [13–15]. Motif identification and analysis have been extensively used to explore these regulatory networks. Network motifs are recurring short patterns in the network. One of the most overrepresented network motif is Feed Forward Loop (FFL) [16]. It is tripartite co-regulatory motif of a Transcription Factor, miRNA and gene in which a TF regulates a miRNA or a miRNA regulates a TF, and both jointly coregulate a target gene. This three-node motifs are found significantly overexpressed in gene regulatory networks [17]. Identification of these motifs has been found helpful in predicting important targets for diagnosis, prognosis, and therapeutic purposes in multifactorial disorders [18]. Recent reports of nSARS-CoV-2 have correlated the pulmonary diseases and thrombosis in terms of similar pathology and symptoms [19–21]. These diseases also cause Acute Lung Injury (ALI) and Acute Respiratory Distress Syndrome (ARDS) that are very common in the critical patients of nSARS-CoV-2. The associations between these diseases have to lead to the possibility of repurposing of drugs that could be effective for treating critical patients [22]. Though there are many published reports on associations in clinical findings or relationships among their pathology and symptoms but the similarity in molecular mechanisms is still not well studied. Analysis of the complex regulatory interactions of these antiviral human miRNAs and their interactions have been used to identify relationships between these diseases and nSARS-CoV-2 at the molecular level.

A list of 11 antiviral human-microRNAs (miRNAs) that can potentially target nSARS-CoV-2 genes was collated [10]. Direct miRNA interactors for these are identified and a comprehensive nSARS-CoV-2 responsive miRNA:TF: gene coregulatory network was built. Overrepresented biological pathways and regulatory FFLs are analyzed and a possible molecular mechanism and checkpoints are proposed. These circuits provide molecular mechanistic insights about nSARS-CoV-2 infection and its similarity with other pulmonary diseases and thromboembolic disorders with interconnections at the molecular level.

## Methodology

### Identification of miRNAs associated with nSARS-CoV-2 antiviral host-miRNAs

The list of 11 antiviral human-microRNAs (miRNAs) that can potentially target nSARS-CoV-2 genes was extracted from Sardar etal study [10]. Nine of these 11 miRNAs- hsa-let-7a, hsa-miR-101, hsa-miR-125a-5p, hsa-miR-126, hsa-miR-222, hsa-miR- 23b, hsa-miR-378, hsa-miR-380-5p and hsa-miR-98 have been identified by them to specifically target nSARS-CoV-2 genome. For this, they fetched verified host antiviral miRNAs with their targets from the VIRmiRNA database and further identified potential host microRNA target sites in the nSARS-CoV-2 virus genome sequences (with a binding energy threshold of -20 kcal/mol). Additionally, they performed an integrated sequence-based analysis of nSARS-CoV-2 genomes from different geographical locations that revealed two host-miRNAs hsa-miR-27b-3p and hsa-miR-27b-5p that target the India specific nSARS-CoV-2 virus genome. This leads to the total 11 miRNAs. Their direct miRNA interactors were identified from PmmR database (http://www.isical.ac.in/∼bioinfo_miu/pmmr.php) [23]. PmmR is a database of putative miRNA-miRNA interactions identified by analysing miRNA:TF: inter-regulatory networks with the help of shortest path length as a scoring function. To make the results more stringent, interactions having a score > 0.7 were only considered. This yielded an exhaustive list of 58 miRNAs which was further used to identify target genes.

### Construction of miRNA:TF: gene co-regulatory network and its pathway enrichment

The miRNA: target genes were identified using the MultimiR R package (http://multimir.ucdenver.edu/) [24]. The package collects data from several validated databases, including mirTarBase, miRecords, and tarbase, etc. These three databases are well-accepted databases for retrieving experimentally validated miRNA-target interactions that have been validated using a different combination of genomics, transcriptomics, and proteomics experiments. Further, a comprehensive list of all human TFs was collated from several databases i.e. TFcheckpoint (www.tfcheckpoint.org) [25], DBD (http://www.transcriptionfactor.org/index.cgi? Home) [26], ORFeome [27], TcoF-DB V2 (https://tools.sschmeier.com/tcof/home/) [28], TFCat (http://www.tfcat.ca) [29], TFClass (http://tfclass.bioinf.med.uni-goettingen.de/) [30] TRANSFAC (http://genexplain.com/transfac/) [31]. The miRNA target genes were compared with this comprehensive TF list and those matching were labelled as TFs and others were labelled as genes. Thus the resultant miRNA: target gene interactions were annotated as miRNA: gene or miRNA: TF interactions. TF:gene and TF:miRNA interactions were also added from respective public repositories. The TF:gene interactions were fetched from oregAnno 3.0 (http://www.oreganno.org/) [32] and TRRUST V2 (https://www.grnpedia.org/trrust/) [33] databases. TF:miRNA interactions were fetched from TransmiR (http://www.cuilab.cn/transmir) [34] and PuTmiR (https://www.isical.ac.in/~bioinfo_miu/TF-miRNA/TF-miRNA.html) [35] databases. Finally a comprehensive nSARS-CoV-2 responsive miRNA: TF: gene coregulatory network was built. Reactome pathway analyser is a powerful tool to mine signalling pathways of genes sets (https://reactome.org/) [36]. It was used to identify the enriched pathways in the TFs and genes of nSARS-CoV-2 responsive miRNA:TF:gene regulatory network. Further the enriched pathways were analysed by boxplot for identification of outliers.

### Identification of miRNA:TF: gene co-regulatory FFL motifs

Among the various types of motifs that can be potentially identified from a network, a randomization test was performed to evaluate the significance of the FFLs. For this, how often one FFL appears in the real network to the number of times it appears in randomly generated networks formed by degree preserving randomization algorithm is compared. To retain biological key driver nodes, a degree preserving the randomization algorithm of the ‘igraph’ R-package is used [37]. The randomization process is repeated 100 times. Z score for each motif type was calculated to identify the significant motifs.

$$ Zscore= No-\mathrm{Nm}/\sigma $$

No is the number of motifs observed in the real network, whereas Nm and σ are the mean and standard deviation of the motif occurrence in 100 random networks, respectively. Thereafter, a tripartite motif (FFL) consisting of a TF, gene and miRNA where either the TF or miRNA or both control a target gene were identified from miRNA:TF: gene coregulatory network using in-house python scripts based on graph theory principle.

## Results and Discussion

Forty-seven miRNAs interacting with 11 antiviral host-miRNAs are identified using the PmmR database (Supplementary Table S1). ~ 5000 experimentally validated gene targets of these 58 human miRNAs were identified. These targets were annotated as TFs or genes by comparing them with a comprehensive list of human TFs. Hence miRNA: target interactions were categorised as miRNA: TF and miRNA: gene interactions. Additionally TF: gene, TF:miRNA interactions were added to construct the comprehensive nSARS-CoV-2 responsive miRNA:TF: gene coregulatory network. This network contains 58 miRNAs, 300 TFs, 4696 genes. (Supplementary Figure S1).

**Fig. 1:**
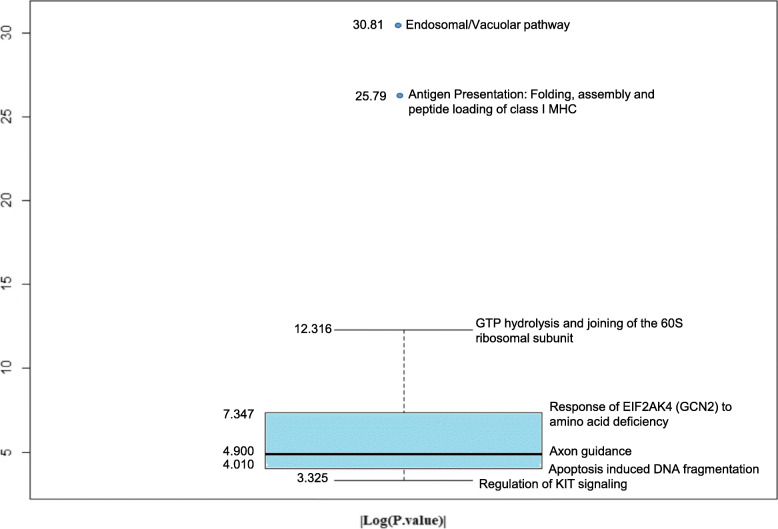
The quartile distribution of enriched pathway clusters based on |log_2_ (*p*Value)|

The TF and genes in the comprehensive nSARS-CoV-2 responsive miRNA:TF: gene coregulatory network were subjected to pathway enrichment using the Reactome pathway analyser [36]. Sixty-eight pathways having *p*. Value < 0.1 were identified (Supplementary Table S2). To identify the most enriched pathways in the network, these pathways terms were distributed based on the modulus of log_2_ (*p*.Value) using boxplot (Fig. 1).

“Endosomal/Vacuolar pathway (R-HSA-1236977)” and “Antigen Presentation: Folding, assembly, and peptide loading for MHC class 1 (R-HSA-983170)” were identified as outliers. Also, the endosomal/vacuolar pathway, also known as Endocytic Pathway, has been recently reported as a major targeting pathway for designing COVID-19 therapeutics because it controls key elements in viral infection i.e. process of viral entry into the host cells [38]. The endocytic pathway including endosome and lysosome has been used for the development of therapeutic strategies in combating diseases caused by other CoVs i.e., SARS, MERS [38]. Similarly, the second most enriched pathway in the network namely “Antigen Presentation: Folding, assembly, and peptide loading for MHC class 1” pathway is reported to affect the susceptibility and severity of nSARS-CoV-2 virus infection in the patients [39].

In a parallel study, a list of 82 genes related to COVID-19, SARS and MERS were curated from literature by text-mining and manual curation (Supplementary Table S3). Further, their associated regulatory miRNAs, TFs, and related interaction were retrieved, and an analogous miRNA:TF: gene coregulatory network was constructed. Pathway enrichment analysis of this network also shows the “Endosomal/Vacuolar signalling pathway” and “Antigen Presentation: Folding, assembly, and peptide loading for MHC class 1” as enriched pathways (Supplementary Table S4). This cross-validates the results i.e. if we begin with host antiviral miRNAs or nSARS-CoV-2 responsive gene list, these two pathways are the most enriched. Hence TFs and genes associated with these pathways were chosen for further analysis.

### Feed Forward Loop analysis

**Fig. 2:**
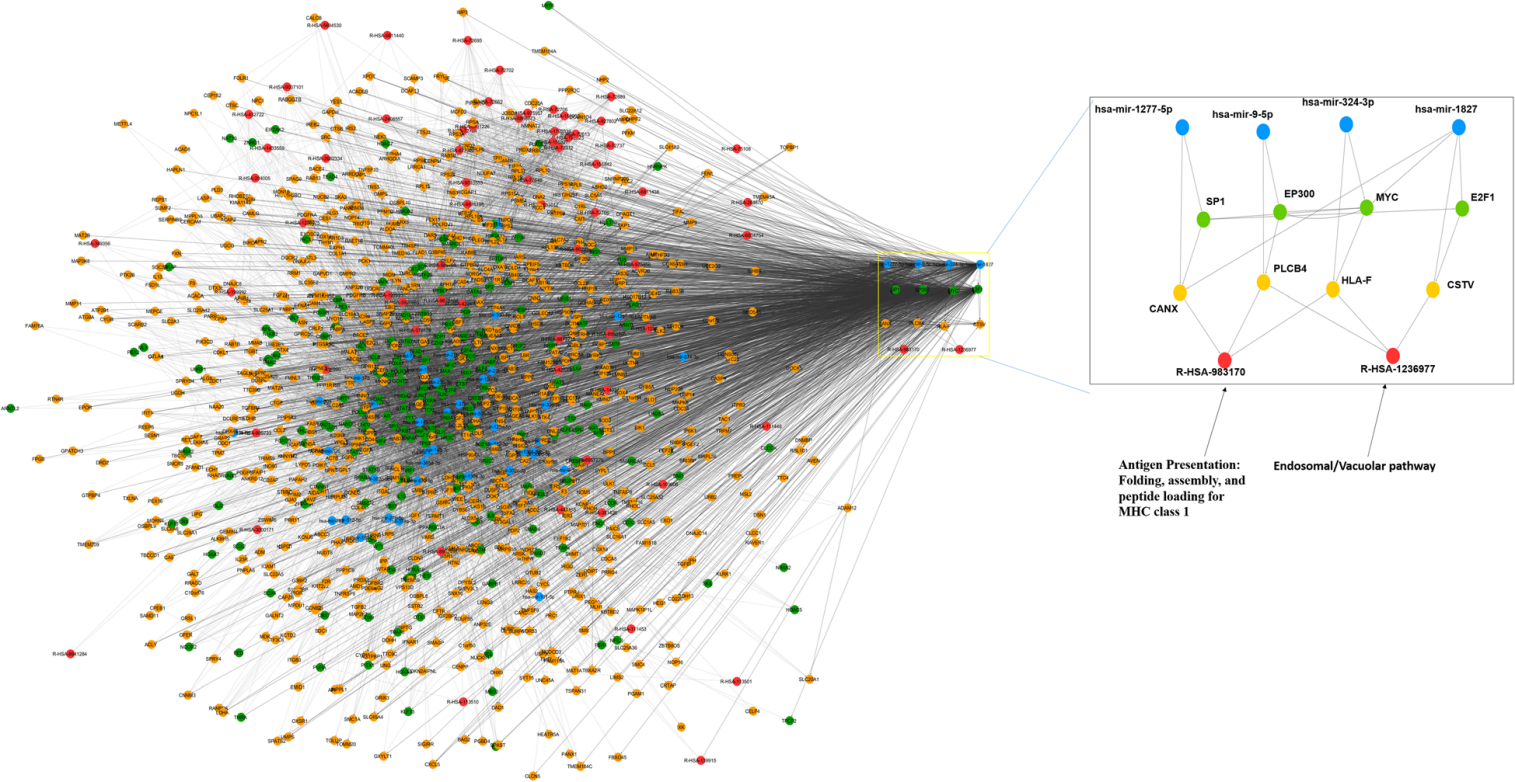
The FFL network identified from miRNA:TF: gene coregulatory network. The miRNAs, TFs, genes, and Reactome pathways are represented in blue, green, yellow, and red nodes respectively. The yellow box contains FFL motifs that are known to regulate/control most enriched pathways of the network i.e. “Endosomal/Vacuolar pathway” (R-HSA-1236977) and “Antigen Presentation: Folding, assembly, and peptide loading for MHC class 1” (R-HSA-983170)

TFs and miRNAs are the two most important key regulators of gene expression at the transcriptional and posttranscriptional levels. Evidence from the literature has suggested the importance of their complex combinatorial regulation in various cellular systems and diseases. miRNAs and TFs are part of a complex combinatorial regulatory mechanism that can be seen in the miRN:TF: gene coregulatory network. These combinatorial regulations can be easily understood by studying miRNA, TF, gene coregulatory networks motifs, which are overrepresented in their coregulatory networks [40, 41]. Network motifs are simple-subgraphs that recur in complex networks. Four different subgraphs (motifs) were identified in the nSARS-CoV-2 responsive miRNA:TF: gene coregulatory network. Randomization was performed for 100 random networks to calculate the Z-score for each type of subgraph. Randomization of network shows motif 000100110 has the highest Z-score of − 0.907 (Supplementary Table S5). The 000100110 type subgraph are FFLs motifs i.e. tripartite motifs consisting of a miRNA, TF and gene coregulating each other. 1385 FFLs were identified and a resultant network contained 17 miRNAs, 101 TFs, 84 gene targets (Fig. 2). Four of these FFLs contained TFs and genes associated with “Endosomal/Vacuolar pathway (R-HSA-1236977)” and “Antigen Presentation: Folding, assembly, and peptide loading for MHC class 1 (R-HSA-983170)” pathways. These FFLs governs most of the interaction traffic in the network; as can be seen from the edge concentration (Fig. 2). This distinct feature helps to identfy hub/important nodes of the biological networks.

Four FFLs are ‘A’: hsa-mir-9-5p-EP300-PLCB4, ‘B’: hsa-mir-324-3p-MYC-HLA-F, ‘C’: hsa-mir-1827-E2F1-CTSV and ‘D’: hsa-mir-1277-5p-SP1-CANX (Table 1). FFL ‘A’ and ‘B’ are common to both the pathways; whereas FFL ‘C’ and ‘D’ are unique to “Endosomal/Vacuolar pathway” and “Antigen Presentation: Folding, assembly, and peptide loading for MHC class 1” respectively.

**Table 1:** The FFL motifs identified in nSARS-CoV-2 responsive miRNA:TF: gene coregulatory network having its target genes or TFs in the enriched pathways

FFL	miRNA	TF	Gene	Pathways
A	hsa-mir-9-5p	EP300	PLCB4	Endosomal/Vacuolar pathway (R-HSA-1236977), Antigen Presentation: Folding, assembly, and peptide loading for MHC class 1 (R-HSA-983170)
B	hsa-mir-324-3p	MYC	HLA-F	Endosomal/Vacuolar pathway (R-HSA-1236977), Antigen Presentation: Folding, assembly, and peptide loading for MHC class 1 (R-HSA-983170)
C	hsa-mir-1827	E2F1	CTSV	Endosomal/Vacuolar pathway (R-HSA-1236977)
D	hsa-mir-1277-5p	SP1	CANX	Antigen Presentation: Folding, assembly, and peptide loading for MHC class 1 (R-HSA-983170)

**Fig. 3:**
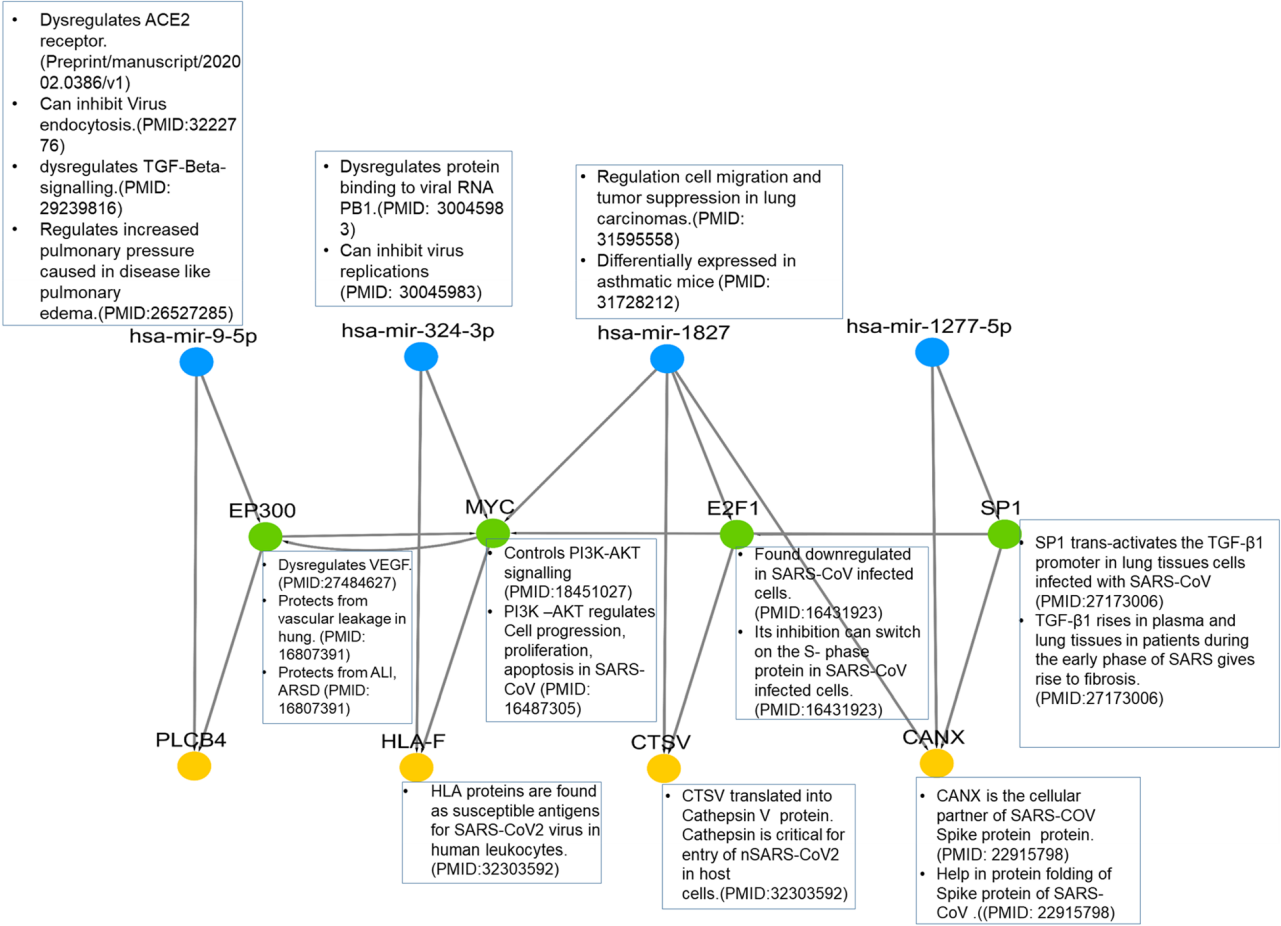
The miRNA-gene regulatory circuits and interactions between them. miRNAs, TFs and genes are represented in blue, green and yellow nodes respectively. The association of each biomolecule with nSARS-CoV-2 is highlighted in boxes adjacent to them with their respective PMIDs

Although these FFLs do not have any common nodes there are many interconnections between them e.g. FFL ‘A’ is connected to FFL ‘B’ through EP300 regulating MYC and vice-versa. Similarly, FFL ‘C’ is connected to FFL ‘B’ through both E2F1, hsa-miR-1827 regulating MYC (Fig. 3). Hence these FFLs can be referred as miRNA-gene regulatory circuits. This closed circuit is known to regulate/control important biological processes as shown in Fig. 3 and discussed below.

The FFL ‘A’: hsa-mir-9-5p-EP300-PLCB4 contains miRNA- hsa-miR-9-5p coregulating TF-EP300 and gene-PLCB4. Hsa-miR-9-5p was recently reported to target the 3′ UTR of Angiotensin-converting enzyme 2 (ACE2) [42]. ACE2 receptor is identified as the main receptor that binds to the spike protein of the COVID-19 virus and triggers viral RNA endocytosis [43]. It is also identified as an initiator of pro-inflammatory cytokine production, and type I interferon production during SARS-CoV infection [44]. Hence hsa-miR-9-5p can be used as a potential target to regulate endocytosis of nSARS-CoV-2 viral RNA and uncontrolled lung inflammation. In FFL ‘A’: hsa-miR-9-5p is found to regulates E1A Binding Protein P300 (EP300), a known transcriptional co-activator protein of Hypoxia-Inducible Factor-1(HIF-1) [45] and stimulator of Vascular Endothelial Growth Factor (VEGF) [46]. VEGF is already identified as a vascular permeability inducer in severe or critical patients with COVID-19 pneumonia. An anti-VEGF drug Bevacizumab is already in the clinical trial to treat critical patients of COVID-19 with ClinicalTrials.gov Identifier: NCT04275414. Both of these miRNA and TFs regulates a common target known as Phospholipase C Beta 4 (PLCB4) in the FFL A. PLCB4 function in the intracellular transduction of many extracellular signals. But there are no reports of an association between PLCB4 and nSARS-CoV-2, SARS and MERS diseases. As PLCB4 is under tight regulation of hsa-miR-9-5p and EP300, which regulates important molecular functions during nSARS-CoV-2 infection, it might be an important novel target for studying its role in nSARS-CoV-2. FFL B hsa-mir-324-3p-MYC-HLA-F, contains hsa-miR-324-3p as a major regulator. Hsa-miR-324-3p was found to inhibit viral replication of RNA viruses like influenza and SARS in host cells by targeting its (Protein Binding 1 to viral RNA) PB1 [47]. In FFL ‘B’, hsa-miR-324-3p deregulates MYC which is a known transcription factor and oncogene controlling cell cycle progression, cell proliferation, and apoptosis [48]. MYC activation also induces the activation of the PI3K–AKT pathway. PI3K –AKT pathway was found as an important pathway that controls virus infected host-cell proliferating genes and possibly helping in viral replication [49, 50]. FFL analysis shows hsa-mir-324-3p and MYC coregulates the HLA-F gene expression. Human leukocyte antigen (HLA) proteins are found as susceptible antigens for nSARS-CoV-2 virus in human leukocytes [39]. Reports also show MYC and hsa-miR-324-3p have an inverted expression profile compared with HLA proteins [51]. This shows HLA-F expression can be regulated by both MYC and hsa-mir-324-3p in human leukocytes and targeting it can enhance the efficacy of the vaccine designed against nSARS-CoV-2. Hence this tightly regulated module where all biomolecules are important during SARS infection could be studied in detail experimentally. miRNAs and TFs of other two unique FFLs ‘C’ and ‘D”: hsa-mir-1827-E2F1- CTSV and hsa-mir-1277-5p- SP1- CANX are also found to regulate two important genes Cathepsin V (CTSV) and Calnexin (CANX). Both of these proteins bind to the Spike protein of coronaviruses of nSARS-CoV-2 and regulate virus entry and stabilizes spike protein for its folding in host cells [6, 52]. The regulatory TFs of FFL ‘C’ and FFL ‘D’ are SP1 and E2F1 respectively. SP1 was reported to trans-activate the TGF-B1 protein in lung tissues during SARS-CoV infection [53]. An increase in the level of TGF-B1 protein in plasma and lungs during SARS-CoV infection gives rise to tissue fibrosis in the lungs. E2F1 was found to downregulate the S-phase genes expression in SARS-CoV infected lung tissue that promotes cell cycle progression and virus replication [54]. As both mechanisms were also found in nSARS-CoV-2, inhibiting E2F1 can weaken the rate of virus replication. Hsa-mir-1827 and hsa-mir-1277-5p are the regulators of FFL ‘C’ and FFL ‘D’. Hsa-mir-1827 was found to regulate tumor suppression in lung carcinomas [55]. There is no evidence of hsa-mir-1277-5p in any pulmonary disease, but they regulate important TFs and genes in the FFLs identified during this analysis. Hence both these miRNAs are proposed as novel targets and open for future experimental analysis and validation. This miRNA-gene circuit with their literature insights has been illustrated in (Fig. 3).

The clinical finding of critical nSARS-CoV-2 patients mainly admitted to intensive care units shows consistent high-level proinflammatory cytokines in their plasma [56, 57]. This cytokine storm was found responsible for causing systemic inflammation that leads to the activation of the too many immune cells such as macrophages, lymphocytes [57]. The increase in the level of cytokines and systematic inflammation were found as the trigger for lung inflammation and coagulation in nSARS-CoV-2 patients [58]. The common symptoms show activation of cytokine storm with consistent fever, cytopenia, enlarged liver or spleen etc. [59]. The variety of cytokines activated during nSARS-CoV-2 infection and the most frequent are interleukins IL2, IL-2R, IL-6, IL1RN, IL-10, IL-7 etc. [56, 60]. Some of these interleukins IL6, IL10, and IL1RN have been also found in the FFLs of nSARS-CoV-2 responsive miRNA:TF: gene coregulatory network (Supplementary Table S7). These interleukins were found to be co-regulated by miRNA, TF i.e. hsa-mir-1277-5p, hsa-mir-98-5p, hsa-mir-149-3p, hsa-mir-125a-5p, hsa-mir-9-5p, hsa-mir-23b-3p, hsa-mir-365a-3p and TFs EP300, FOXO1, PPARA, REL, respectively.

Other inflammatory cytokines like TNF-α, TGF-β that have been found correlated with coagulation and systematic inflammation during nSARS-CoV-2 are also important signaling molecules of other pathologies i.e. lung injury and thrombosis [61].

**Fig. 4:**
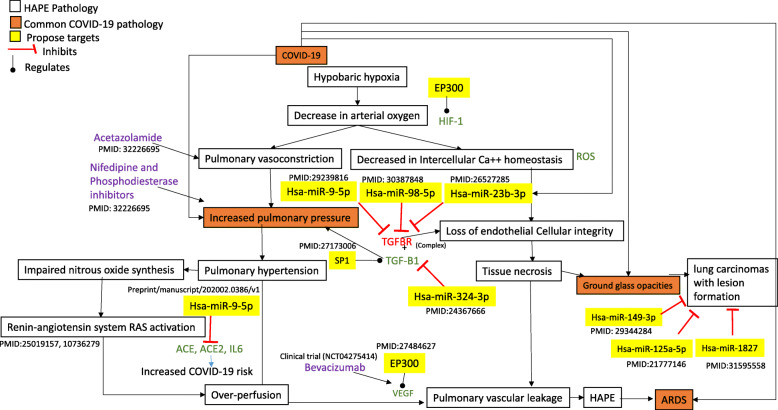
The cohesion in the HAPE and nSARS-CoV-2 infection at both pathophysiological and molecular level with common biomolecular targets and drugs. The white boxes depict the HAPE pathology, orange boxes depicts the common pathologies of HAPE that are seen in nSARS-CoV-2 infection. Yellow boxes depict the biomolecules from the current study which are part of nSARS-CoV-2 miRNA-gene regulatory circuit or their direct interactors. The drugs used in HAPE that are proposed to be repurposed for nSARS-CoV-2 are highlighted in purple text. The reference literature (PMID) which provides the evidence for interactions between the biomolecules are also listed adjacent to them

**Fig. 5:**
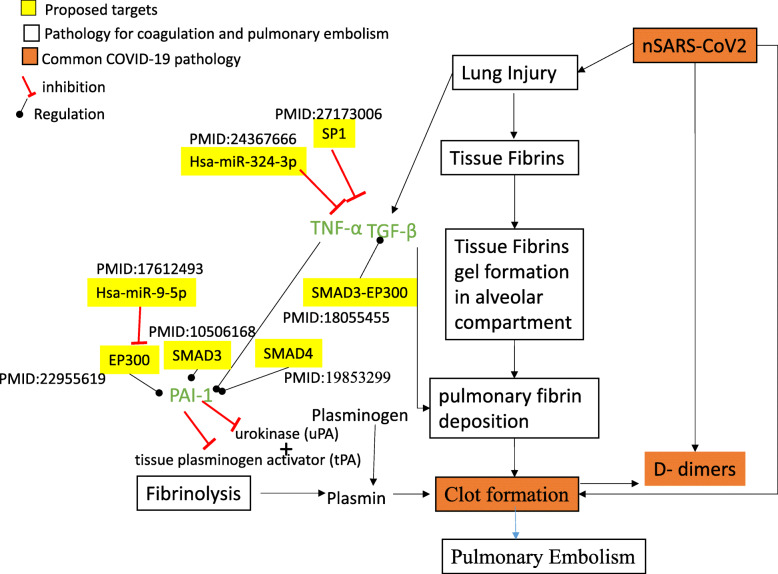
The similarity in the coagulation, pulmonary embolism pathology with nSARS-CoV-2 infection. The white boxes depicts the normal pathology of coagulation and pulmonary embolism, orange boxes depicts the common pathology that are seen in nSARS-CoV-2 infection. Yellow boxes depict the biomolecules from the present study, which are part of miRNA-gene regulatory circuit in nSARS-CoV-2 or their direct regulators. The biomolecules highlighted in green (TNF-α, TGF-β and PA-1) are proposed as main interconnections between nSARS-CoV-2 and pulmonary embolism. The reference literature (PMID) which provides the evidence for interactions between the biomolecules are also listed adjacent to them

Transforming growth factor β (TGF-β) cytokine has a multi-faceted regulatory and inflammatory activity that depends on the cellular type and stress environment [62, 63]. The TGF-β expression found in all types of cells and can suppress or promote inflammation [64]. A recent clinical study shows a massive spike in active TGF-β in nSARS-CoV-2 patients [65]. This study also proposes three reasons for the spike of active TGF-β in nSARS-CoV-2 patient’s lungs. First is the activation of the latent (inactive) TGF-β in the lungs due to immune and inflammatory responses as well as fibrinolytic pathways. This results in the overall decrease in circulating levels of latent TGF-β in patients with pneumonia, as more active TGF-β, is present in the lungs. Second, nSARS-CoV-2 infection increases the infiltration of neutrophils into the lungs. Neutrophils release TGF-β that can further be activated by elastase enzyme of neutrophils and cause a massive increase in the active form of TGF-β in the lungs. Third, apoptosis and necrosis of bronchial epithelial cells (ground-glass opacities in the lungs), pneumocytes, and T lymphocytes takes place during nSARS-CoV-2 infection. This leads to induction and migration of macrophages in the lungs. Macrophages can induce inflammation by secreting large amounts of latent (and active) TGF-β into the lungs that further activates local proteases such as furin, plasmin, and elastase, reactive oxygen species (ROS), Matrix metalloproteinases (MMPs), and integrins [65]. Due to the sudden increase in the active TGF-β lungs with some other proinflammatory cytokines like TNFα, IL-6, and IL-1β causing systematic inflammation leads to edema and fibrosis in the lungs. Whereas TNF-α is a proinflammatory cytokine that induces the inflammatory cascade, resulting in lung injury during SARS-CoV-2 infection [66]. TNF-α inhibition has, in fact, been demonstrated to reduce the severity of virus specific lung immunopathology in mice [66]. Anti-TNF antibody produced a dramatic reduction of overall illness severity without interfering with viral clearance. Hence TNF-α inhibitors are being used to treat the severe and critical patients of nSARS-CoV-2 [67]. miRNA, TF regulators of these inflammatory cytokines found in the nSARS-CoV-2 responsive miRNA:TF: gene coregulatory network has also been found to be highly correlated with nSARS-CoV-2 and their potential correlations with pulmonary diseases and coagulation (Figs. 4 and 5).

Recent reports show that nSARS-CoV infection has a symptom similarity with some of the pulmonary diseases like Acute Lung Injury (ALI), Acute Respiratory Distress Syndrome (ARDS) and Chronic Pulmonary Obstructive Disorder (COPD) [68, 69]. This hints towards the possibility of cohesion in terms of molecular signalling and pathology of pulmonary diseases with nSARS-CoV-2. But there are some contrasting reports for establishing comorbidity in HAPE and nSARS-CoV-2. Some reports favor the similarity in their pathophysiologies [19], whereas other reports decline the similarity as HAPE is non-cardiogenic form of pulmonary edema caused due to hypoxic pulmonary vasoconstriction [70] and support that nSARS-CoV-2 caused lung injury is only due to viral mediated inflammation [70]. Both the studies might be true but they lack the support of molecular evidence. Hence we try to overlay our FFL analysis results and literature finding over well-established HAPE framework (Fig. 4).

For this a list of 93 genes related to HAPE was manually curated from the literature (Supplementary Table S6). Six of these genes/TFs are also present in the FFL network of nSARS-CoV-2. These include PPARA, MAP 2 K7, IL6, IL10, IL1RN and VCAM1. These genes are linked to HAPE as a biomarker or their polymorphism as a risk factor in HAPE-resistive Vs HAPE susceptible patients [71–73]. When these 6 genes were mapped on the FFLs, 15 FFLs were identified and found that most of the miRNAs regulated the TGF-beta signalling and tumor suppression in pulmonary diseases (Supplementary Table S7). Also, the plasma levels of IL6, IL10 and IL1RN were found to increase in the critical nSARS-CoV-2 patients [57]. So systemic inflammation caused due to a strong influx of cytokines like interleukins has a strong association between pulmonary diseases like HAPE and nSARS-CoV-2. Literature also shows increased pulmonary pressure and ground glass opacities were found common in both HAPE and nSARS-CoV-2 patients [19]. During HAPE, hypobaric hypoxia causes a decrease in arterial oxygen leading to the pulmonary vasoconstriction and further leads to an increase in pulmonary pressure. TGF-β signalling was identified to control pulmonary pressure in lungs and TGF-B1 and TGFBR receptors-1,2 complex are the major regulators of the signalling [74]. The sustained increase in pulmonary pressure causes pulmonary hypertension, over perfusion that further leads to pulmonary vascular leakage causing edema. Recently published reports also show that TGF-beta1 appears to be overproduced in COVID-19 patients [69]. Hsa-miR-9-5p, hsa-miR-98-5p and hsa-miR-23b-3p regulate TGFBR receptors during pulmonary disorders [74–76]. These miRNAs are part of the regulatory circuit identified in the present study and are antiviral host-miRNAs that can potentially target nSARS-CoV-2 genes [10]. Also, pulmonary hypertension impairs nitrous oxide synthesis that induces RAS pathways proteins during HAPE. These proteins include IL6, ACE, ACE2 that cause inflammation and over-perfusion leading to pulmonary vascular leakage [77]. ACE2 and its receptors have been widely identified as risk factor for COVID-19 patients. Also, a decrease in arterial oxygen during HAPE leads to a decrease in intercellular Ca^++^ homeostasis marked with ROS generation causing loss of endothelial cellular integrity. This induces tissue necrosis in the lungs seen as ground glass opacities in radiological scans of HAPE and lung carcinoma patients [78, 79]. This loss of endothelial cellular integrity causing necrosis is also controlled by TGF-β signalling [80, 81]. The ground glass opacities is reported in both nSARS-CoV-2 infection and lung carcinomas. miRNAs hsa-miR-149-5p, hsa-miR-125a-5p, hsa-miR-1827 in our 15 FFLs were found to regulate tumor suppression cell migration, invasion and apoptosis in the lung carcinomas with lesion formation [82, 83]. Hence these miRNAs can be studied and their role in tissue necrosis in nSARS-CoV-2 infection can be explored. The literature also highlights commonality in pathophysiology like pulmonary pressure, lung fibrosis in both HAPE and SARS-CoV viral infection [53, 74]. This factor is important in the repurposing of drugs like Acetazolamide, Nifedipine and Phosphodiesterase inhibitors that have been proposed to be repurposed for COVID-19 [19]. Acetazolamide has a myriad of effects on different organ systems, potently reduces hypoxic pulmonary vasoconstriction and improves minute ventilation; whereas Nifedipine and Phosphodiesterase inhibitors are directed to decrease pulmonary pressure in HAPE patients [19].

The miRNAs proposed in the current study were submitted to Pharmaco-miR [84] server to identify their potential interactions with drugs. Nifedipine and Acetazolamide were found to be associated with the expression of hsa-mir-98 and hsa-mir-23b respectively [84]. Further screening of other miRNA, TF, gene targets in network can also be experimentally validated to design therapeutic drugs, diagnosis marker, prognosis marker or vaccine against nSARS-CoV-2.

Recent observations of nSARS-CoV-2 cases also suggest that pulmonary and lung injury in COVID-19 is not the only factor to drive patients to acute respiratory distress syndrome (ARDS) and further to Acute cardiac injury (ACI), Acute Kidney injury (AKI) and multiple organ failure. Other factors like thrombosis and sepsis are also proposed [85]. Activation of coagulation can be easily found in a variety of virus infections like retrovirus outbreak including HIV, Dengue virus, and Ebola virus especially in SARS outbreak of 2003 [86]. Reports suggested that high morbidity and mortality rates of SARS which is closely related to nSARS-CoV-2 is due to the vascular endothelial damage together with Disseminated Intravascular Coagulation (DIC), Deep Vein Thrombosis (DVT) and Pulmonary Embolism (PE) that leads to the pulmonary infarction [87, 88]. The increase of D-dimers in nSARS-CoV-2 patients during nSARS-CoV-2 disease progression with chest CT shows a strong association with venous thrombosis and pulmonary embolism [60]. D-dimers are the product of degraded cross-linked fibrin from the clot formation [89]. Whether the molecular mechanism leading to clot can be virus-mediated or result of systemic inflammation is the grey area of research.

The clot formation is initiated in the most type of lung injuries [90]. Lung injury leads to the formation of fibrin gels inside the alveolar compartment. The fibrin gel is the accumulation of tissue fibrins formed after the disruption of fibrous network around human lung fibroblasts. Due to the lack of fibrin networks around human lung fibroblasts, they escape the cell-matrix and support the organization of these fibrin gels. This growth of fibrin gels further leads to clot formation. After clot formation, fibrinolysis is a process that prevents blood clots from growing. During fibrinolysis, plasminogen is reduced to plasmin by tissue plasminogen activator (tPA) and urokinase (uPA) (Fig. 5).

Plasmin then degrades the clot fibrin network into smaller products and reduces further clot formation [91]. HLF plasminogen activator inhibitor-1 (PAI-1) or SERPINE1 is identified as an inhibitor of tPA and uPA. Inhibition of tPA and uPA leads to the inactivation of plasmin enzyme that further prevents the process of fibrinolysis [91]. Literature shows that lung injury also induces cytokines like TGF-β and TNF-α. TGF-β and TNF-α have been extensively studied in Human Lung Fibroblasts (HLF) for their role in fibrinolytic and procoagulant activities. TGF-β help in the induction HLF plasminogen activator inhibitor-1 (PAI-1), Human Coagulation Factor XII in human lung fibroblast and TNF-α induces release of PAI-1 from the cells and inactivates tPA [91, 92]. This way, TGF-β and TNF-α impair the ability of HLF to degrade clot fibrin by disturbing the balance of HLF plasminogen activators and PAI-1. These pathways may therefore be targeted for a possible mechanism to protect patient from pulmonary embolism and thrombosis.

It was found that nucleocapsid (N) protein potentiates TGF-β -induced expression of PAI-1 during SARS-CoV infection [93]. The PAI-1 or SERPINE1 is regulated by many TFs in nSARS-CoV-2 responsive miRNA: TF:gene coregulatory network (Supplementary Table S8). Some of these TFs i.e. SMAD-3,SMAD4, EP300 have been found modulated by N protein of SARS-CoV virus [93]. N protein binds to SMAD3/SMAD4 of host cell and promotes SMAD3-EP300 complex formation that regulates the TGF-β signalling pathway during SARS-CoV infection [93]. So hsa-miR-9-5p that dysregulates the EP300 in the proposed miRNA-TF-gene regulatory circuit can also be used as target in nSARS-CoV-2 [94]. Interleukin IL6 induction was also seen in the later stage nSARS-CoV-2 patients [60]. It was also seen that IL-6 has opposite expression profile as compared with D-dimer in nSARS-CoV-2 patients. Its elevation starts from 13 days onwards of the infection as when D-dimers start to decrease [60]. IL-6 promotes coagulation without affecting fibrinolysis [95]. Hence increase in IL6 and decrease in D-dimer might be an indicator of inhibition of fibrinolysis, which can further leads to the growth of clot to the extent causing serious thrombotic conditions.

## Conclusion

Our study has identified four miRNAs (hsa-mir-9-5p, hsa-mir-324-3p, hsa-mir-1827, and hsa-mir-1277-5p) and TFs (EP300, MYC, E2F1, SP1) that co-regulate important target genes responsible for nSARS-CoV-2 viral endocytosis, viral replication and its antigen presentation in the host system. The study also helps in finding common molecular trajectories of nSARS-CoV-2 with clotting, pulmonary embolism, pulmonary edema, and systematic inflammation which shows fibrinolysis as a major pathway that increases the D-dimers in the nSARS-CoV-2 infection and cytokines TGF-β and TNF-α as major regulators of fibrinolysis controlling proteins PAI-1 and plasminogen activators. The host miRNAs and TFs that are part nSARS-CoV-2 FFL network and controlling TGF-β signaling have been proposed as potential target candidates for further experimental investigation.

## Supplementary information

**Additional file 1: Figure S1.** Network representation of comprehensive nSARS-CoV2 responsive miRNA:TF:gene coregulatory directed tripartite network. The miRNAs, TFs and genes are denoted in blue, green and yellow colour nodes respectively. **Table S1.** The list of interactions between the 11 antiviral host miRNAs (highlighted in red) and their direct miRNA indicators. **Table S2.** The pathway enrichment of TFs and genes present in comprehensive nSARS-CoV2 responsive miRNA:TF:gene coregulatory network. **Table S3.** A comprehensive list of genes related to β-coronavirus extracted from literature using textmining and manual curation. **Table S4.** The top 10 enriched pathways miRNA-TF-gene coregulatory network that was constructed using 82 β-coronavirus genes curated from the literature. **Table S5.** Z-scores of different subgraphs calculated after randomization of miRNA-TF-gene-coregulatory network. **Table S6.** The list of the genes expressed or regulated during High altitude pulmonary edema. **Table S7.** The list of the 15 FFLs present in miRNA-TF-gene coregulatory network that are having common HAPE TFs/genes. **Table S8.** The list of TFs regulating SERPINE1 in miRNA-TF-gene coregulatory network.

## Data Availability

All data generated or analysed during this study are included in this published article (and its supplementary information files).

## Abbreviations

nSARS-CoV-2: Novel Severe Acute Respiratory Syndrome Coronavirus 2
SARS: Severe Acute Respiratory Syndrome
TF: Transcription Factor
TG: Target Gene
FFL: Feed Forward Loop
HAPE: High Altitude Pulmonary Edema
ALI: Acute Lung Injury
ARDS: Acute Respiratory Distress Syndrome
