# Supplementary material for: nSARS-Cov-2, pulmonary edema and thrombosis: possible molecular insights using miRNA-gene circuits in regulatory networks
Source: ExRNA. 2020 Oct 30;2(1):16. doi: 10.1186/s41544-020-00057-y (PMC7596315; doi:10.1186/s41544-020-00057-y)
Supplement: Supplementary file 1 — Additional file 1: Figure S1. Network representation of comprehensive nSARS-CoV2 responsive miRNA:TF:gene coregulatory directed tripartite network. The miRNAs, TFs and genes are denoted in blue, green and yellow colour nodes respectively. Table S1. The list of interactions between the 11 antiviral host miRNAs (highlighted in red) and their direct miRNA indicators. Table S2. The pathway enrichment of TFs and genes present in comprehensive nSARS-CoV2 responsive miRNA:TF:gene coregulatory network. Table S3. A comprehensive list of genes related to β-coronavirus extracted from literature using textmining and manual curation. Table S4. The top 10 enriched pathways miRNA-TF-gene coregulatory network that was constructed using 82 β-coronavirus genes curated from the literature. Table S5. Z-scores of different subgraphs calculated after randomization of miRNA-TF-gene-coregulatory network. Table S6. The list of the genes expressed or regulated during High altitude pulmonary edema. Table S7. The list of the 15 FFLs present in miRNA-TF-gene coregulatory network that are having common HAPE TFs/genes. Table S8. The list of TFs regulating SERPINE1 in miRNA-TF-gene coregulatory network. [file 41544_2020_57_MOESM1_ESM.docx]

**Figure S1: Network representation of comprehensive nSARS-CoV2 responsive miRNA:TF:gene coregulatory directed tripartite network. The miRNAs, TFs and genes are denoted in blue, green and yellow colour nodes respectively.**

**Table S1: The list of interactions between the 11 antiviral host miRNAs (highlighted in red) and their direct miRNA indicators**

| **miRNA 1** | **miRNA 2** | **Score** |
| --- | --- | --- |
| hsa-mir-125a-5p | hsa-mir-598 | 0.861411 |
| hsa-mir-125a-5p | hsa-mir-619 | 0.853321 |
| hsa-mir-125a-5p | hsa-mir-33b | 0.851702 |
| hsa-mir-101 | hsa-mir-1304 | 0.844639 |
| hsa-mir-125a-5p | hsa-mir-591 | 0.840376 |
| hsa-mir-125a-5p | hsa-mir-302a | 0.837139 |
| hsa-mir-125a-5p | hsa-mir-302b | 0.837139 |
| hsa-mir-125a-5p | hsa-mir-302c | 0.837139 |
| hsa-mir-125a-5p | hsa-mir-302d | 0.837139 |
| hsa-mir-125a-5p | hsa-mir-367 | 0.837139 |
| hsa-mir-508-5p | hsa-mir-98 | 0.794808 |
| hsa-mir-323-5p | hsa-mir-98 | 0.790961 |
| hsa-mir-380-5p | hsa-mir-1292 | 0.783824 |
| hsa-mir-891a | hsa-mir-98 | 0.783198 |
| hsa-mir-380-5p | hsa-mir-670 | 0.770879 |
| hsa-mir-574-3p | hsa-mir-98 | 0.769145 |
| hsa-mir-27b | hsa-mir-1275 | 0.758382 |
| hsa-mir-125a-5p | hsa-mir-566 | 0.757848 |
| hsa-mir-125a-5p | hsa-mir-762 | 0.756229 |
| hsa-mir-125a-5p | hsa-mir-1277 | 0.754611 |
| hsa-mir-101 | hsa-mir-1537 | 0.754024 |
| hsa-mir-125a-5p | hsa-mir-512-3p | 0.748139 |
| hsa-mir-125a-5p | hsa-mir-512-5p | 0.748139 |
| hsa-mir-125a-5p | hsa-mir-608 | 0.743284 |
| hsa-mir-668 | hsa-mir-23b | 0.74217 |
| hsa-mir-380-5p | hsa-mir-9 | 0.740134 |
| hsa-mir-125a-5p | hsa-mir-553 | 0.740048 |
| hsa-mir-27b | hsa-mir-197 | 0.735727 |
| hsa-mir-27b | hsa-mir-542 | 0.73434 |
| hsa-mir-125a-5p | hsa-mir-1287 | 0.733576 |
| hsa-mir-365 | hsa-mir-23b | 0.731176 |
| hsa-mir-532-5p | hsa-mir-23b | 0.727573 |
| hsa-mir-208a | hsa-mir-98 | 0.722517 |
| hsa-mir-208b | hsa-mir-98 | 0.722517 |
| hsa-mir-149 | hsa-mir-98 | 0.722364 |
| hsa-mir-380-5p | hsa-mir-1308 | 0.719098 |
| hsa-mir-27b | hsa-mir-549 | 0.718159 |
| hsa-mir-125a-5p | hsa-mir-375 | 0.717395 |
| hsa-mir-324-3p | hsa-mir-98 | 0.716364 |
| hsa-mir-149 | hsa-mir-23b | 0.714549 |
| hsa-mir-27b | hsa-mir-1827 | 0.713304 |
| hsa-mir-101 | hsa-mir-549 | 0.711777 |
| hsa-mir-101 | hsa-mir-152 | 0.708717 |
| hsa-mir-193a-5p | hsa-mir-23b | 0.708672 |
| hsa-mir-587 | hsa-mir-23b | 0.706959 |
| hsa-mir-27b | hsa-mir-219-1-5p | 0.706831 |
| hsa-mir-101 | hsa-mir-219-1-5p | 0.70045 |
| hsa-mir-326 | hsa-mir-378 | 0.7 |
| hsa-mir-330-5p | hsa-mir-378 | 0.7 |
| hsa-mir-486-5p | hsa-mir-378 | 0.7 |
| hsa-mir-638 | hsa-mir-378 | 0.7 |
| hsa-let-7a | hsa-mir-100 | 0.7 |

**Table S2: The pathway enrichment of TFs and genes present in comprehensive nSARS-CoV2 responsive miRNA:TF:gene coregulatory network.**

| Pathway identifier | Pathway name | Entities pValue | \|log(p.Value)\| | Entities FDR | Species identifier |
| --- | --- | --- | --- | --- | --- |
| R-HSA-1236977 | Endosomal/Vacuolar pathway | 5.31E-10 | 30.81057 | 1.23E-06 | 9606 |
| R-HSA-983170 | Antigen Presentation: Folding, assembly and peptide loading of class I MHC | 1.72E-08 | 25.79302 | 1.99E-05 | 9606 |
| R-HSA-72706 | GTP hydrolysis and joining of the 60S ribosomal subunit | 1.96E-04 | 12.31686 | 0.151120903 | 9606 |
| R-HSA-156827 | L13a-mediated translational silencing of Ceruloplasmin expression | 3.08E-04 | 11.66478 | 0.175144121 | 9606 |
| R-HSA-72689 | Formation of a pool of free 40S subunits | 3.78E-04 | 11.36933 | 0.175144121 | 9606 |
| R-HSA-72613 | Eukaryotic Translation Initiation | 5.44E-04 | 10.84411 | 0.179632393 | 9606 |
| R-HSA-72737 | Cap-dependent Translation Initiation | 5.44E-04 | 10.84411 | 0.179632393 | 9606 |
| R-HSA-6791226 | Major pathway of rRNA processing in the nucleolus and cytosol | 9.55E-04 | 10.03221 | 0.276044968 | 9606 |
| R-HSA-156902 | Peptide chain elongation | 0.001579 | 9.306666 | 0.371358783 | 9606 |
| R-HSA-975956 | Nonsense Mediated Decay (NMD) independent of the Exon Junction Complex (EJC) | 0.001668 | 9.227811 | 0.371358783 | 9606 |
| R-HSA-1236974 | ER-Phagosome pathway | 0.001768 | 9.14336 | 0.371358783 | 9606 |
| R-HSA-156842 | Eukaryotic Translation Elongation | 0.002087 | 8.904286 | 0.400722908 | 9606 |
| R-HSA-199991 | Membrane Trafficking | 0.002661 | 8.553967 | 0.457589741 | 9606 |
| R-HSA-1799339 | SRP-dependent cotranslational protein targeting to membrane | 0.002961 | 8.399881 | 0.457589741 | 9606 |
| R-HSA-909733 | Interferon alpha/beta signaling | 0.002971 | 8.39466 | 0.457589741 | 9606 |
| R-HSA-8868773 | rRNA processing in the nucleus and cytosol | 0.003407 | 8.197291 | 0.490605026 | 9606 |
| R-HSA-9633012 | Response of EIF2AK4 (GCN2) to amino acid deficiency | 0.006089 | 7.359487 | 0.800333902 | 9606 |
| R-HSA-1236975 | Antigen processing-Cross presentation | 0.006294 | 7.311886 | 0.800333902 | 9606 |
| R-HSA-72764 | Eukaryotic Translation Termination | 0.007029 | 7.152389 | 0.800333902 | 9606 |
| R-HSA-927802 | Nonsense-Mediated Decay (NMD) | 0.007458 | 7.066963 | 0.800333902 | 9606 |
| R-HSA-975957 | Nonsense Mediated Decay (NMD) enhanced by the Exon Junction Complex (EJC) | 0.007458 | 7.066963 | 0.800333902 | 9606 |
| R-HSA-8873719 | RAB geranylgeranylation | 0.007622 | 7.035572 | 0.800333902 | 9606 |
| R-HSA-376176 | Signaling by ROBO receptors | 0.009315 | 6.746178 | 0.829797198 | 9606 |
| R-HSA-72649 | Translation initiation complex formation | 0.016944 | 5.883101 | 0.829797198 | 9606 |
| R-HSA-72702 | Ribosomal scanning and start codon recognition | 0.017267 | 5.855833 | 0.829797198 | 9606 |
| R-HSA-199992 | trans-Golgi Network Vesicle Budding | 0.019086 | 5.711351 | 0.829797198 | 9606 |
| R-HSA-8950505 | Gene and protein expression by JAK-STAT signaling after Interleukin-12 stimulation | 0.022365 | 5.482594 | 0.829797198 | 9606 |
| R-HSA-72695 | Formation of the ternary complex, and subsequently, the 43S complex | 0.023993 | 5.381234 | 0.829797198 | 9606 |
| R-HSA-72662 | Activation of the mRNA upon binding of the cap-binding complex and eIFs, and subsequent binding to 43S | 0.026213 | 5.253584 | 0.829797198 | 9606 |
| R-HSA-983169 | Class I MHC mediated antigen processing & presentation | 0.027409 | 5.189188 | 0.829797198 | 9606 |
| R-HSA-447115 | Interleukin-12 family signaling | 0.027736 | 5.172101 | 0.829797198 | 9606 |
| R-HSA-428359 | Insulin-like Growth Factor-2 mRNA Binding Proteins (IGF2BPs/IMPs/VICKZs) bind RNA | 0.02985 | 5.066104 | 0.829797198 | 9606 |
| R-HSA-432722 | Golgi Associated Vesicle Biogenesis | 0.031066 | 5.00854 | 0.829797198 | 9606 |
| R-HSA-2408557 | Selenocysteine synthesis | 0.031586 | 4.984571 | 0.829797198 | 9606 |
| R-HSA-422475 | Axon guidance | 0.035514 | 4.81546 | 0.829797198 | 9606 |
| R-HSA-418990 | Adherens junctions interactions | 0.0373 | 4.744669 | 0.829797198 | 9606 |
| R-HSA-8876198 | RAB GEFs exchange GTP for GDP on RABs | 0.03827 | 4.707656 | 0.829797198 | 9606 |
| R-HSA-5694530 | Cargo concentration in the ER | 0.038488 | 4.699454 | 0.829797198 | 9606 |
| R-HSA-9007101 | Rab regulation of trafficking | 0.03976 | 4.652533 | 0.829797198 | 9606 |
| R-HSA-3928665 | EPH-ephrin mediated repulsion of cells | 0.044704 | 4.483462 | 0.829797198 | 9606 |
| R-HSA-109606 | Intrinsic Pathway for Apoptosis | 0.045522 | 4.457301 | 0.829797198 | 9606 |
| R-HSA-204005 | COPII-mediated vesicle transport | 0.045936 | 4.444231 | 0.829797198 | 9606 |
| R-HSA-192823 | Viral mRNA Translation | 0.048364 | 4.36992 | 0.829797198 | 9606 |
| R-HSA-9010553 | Regulation of expression of SLITs and ROBOs | 0.049253 | 4.343658 | 0.829797198 | 9606 |
| R-HSA-72766 | Translation | 0.049406 | 4.339161 | 0.829797198 | 9606 |
| R-HSA-111448 | Activation of NOXA and translocation to mitochondria | 0.049691 | 4.330886 | 0.829797198 | 9606 |
| R-HSA-8952158 | RUNX3 regulates BCL2L11 (BIM) transcription | 0.049691 | 4.330886 | 0.829797198 | 9606 |
| R-HSA-6811438 | Intra-Golgi traffic | 0.052864 | 4.241567 | 0.829797198 | 9606 |
| R-HSA-9020591 | Interleukin-12 signaling | 0.053361 | 4.228067 | 0.829797198 | 9606 |
| R-HSA-111453 | BH3-only proteins associate with and inactivate anti-apoptotic BCL-2 members | 0.05482 | 4.189141 | 0.829797198 | 9606 |
| R-HSA-140342 | Apoptosis induced DNA fragmentation | 0.06205 | 4.010432 | 0.829797198 | 9606 |
| R-HSA-113501 | Inhibition of replication initiation of damaged DNA by RB1/E2F1 | 0.06205 | 4.010432 | 0.829797198 | 9606 |
| R-HSA-9675108 | Nervous system development | 0.062353 | 4.003389 | 0.829797198 | 9606 |
| R-HSA-113510 | E2F mediated regulation of DNA replication | 0.062722 | 3.994877 | 0.829797198 | 9606 |
| R-HSA-6811440 | Retrograde transport at the Trans-Golgi-Network | 0.065147 | 3.940149 | 0.829797198 | 9606 |
| R-HSA-381426 | Regulation of Insulin-like Growth Factor (IGF) transport and uptake by Insulin-like Growth Factor Binding Proteins (IGFBPs) | 0.067189 | 3.895638 | 0.829797198 | 9606 |
| R-HSA-8957275 | Post-translational protein phosphorylation | 0.073877 | 3.758733 | 0.829797198 | 9606 |
| R-HSA-72312 | rRNA processing | 0.074295 | 3.750599 | 0.829797198 | 9606 |
| R-HSA-139915 | Activation of PUMA and translocation to mitochondria | 0.074597 | 3.744748 | 0.829797198 | 9606 |
| R-HSA-877300 | Interferon gamma signaling | 0.07858 | 3.669703 | 0.829797198 | 9606 |
| R-HSA-264870 | Caspase-mediated cleavage of cytoskeletal proteins | 0.083224 | 3.586863 | 0.829797198 | 9606 |
| R-HSA-8941284 | RUNX2 regulates chondrocyte maturation | 0.088394 | 3.499906 | 0.829797198 | 9606 |
| R-HSA-3000171 | Non-integrin membrane-ECM interactions | 0.091114 | 3.456183 | 0.829797198 | 9606 |
| R-HSA-389356 | CD28 co-stimulation | 0.093293 | 3.422087 | 0.829797198 | 9606 |
| R-HSA-6804754 | Regulation of TP53 Expression | 0.099169 | 3.333961 | 0.829797198 | 9606 |
| R-HSA-75108 | Activation, myristolyation of BID and translocation to mitochondria | 0.099169 | 3.333961 | 0.829797198 | 9606 |
| R-HSA-2682334 | EPH-Ephrin signaling | 0.099598 | 3.327738 | 0.829797198 | 9606 |
| R-HSA-1433559 | Regulation of KIT signaling | 0.099778 | 3.325129 | 0.829797198 | 9606 |

**Table S3: A comprehensive list of genes related to β-coronavirus extracted from literature using textmining and manual curation.**

| **Rank** | **GeneID** | **Symbol** | **n** | **FDR** | **Top 10 pmids** |
| --- | --- | --- | --- | --- | --- |
| 1 | 59272 | ACE2 | 235 | 1.76E-58 | 24172901 (p=4.42e-05) 18448527 (p=8.25e-05) 17079315 (p=8.39e-05) 15897467 (p=9.07e-05) 14647384 (p=9.18e-05) 16001071 (p=9.31e-05) 24227843 (p=9.43e-05) 20484496 (p=9.48e-05) 22291007 (p=9.79e-05) 15791205 (p=9.80e-05) |
| 2 | 7113 | TMPRSS2 | 161 | 7.97E-14 | 27733646 (p=7.16e-05) 24027332 (p=8.56e-05) 24227843 (p=9.43e-05) 21325420 (p=1.01e-04) 26379044 (p=2.00e-04) 21068237 (p=6.04e-04) 23536651 (p=6.04e-04) 22558251 (p=9.01e-04) 21123387 (p=9.03e-04) 23192872 (p=1.30e-03) |
| 3 | 23586 | DDX58 | 349 | 4.16E-12 | 28148787 (p=8.02e-05) 28659477 (p=1.04e-04) 26311867 (p=1.50e-03) 24850742 (p=2.30e-03) 27122586 (p=4.00e-03) 24371060 (p=4.50e-03) 25552708 (p=5.50e-03) 19374189 (p=6.80e-03) 26378160 (p=8.10e-03) 25833049 (p=8.90e-03) |
| 4 | 10332 | CLEC4M | 105 | 4.51E-12 | 17715238 (p=9.60e-05) 15496474 (p=9.90e-05) 18697825 (p=1.03e-04) 23788638 (p=3.02e-04) 17632570 (p=7.01e-04) 16369534 (p=1.60e-03) 18708672 (p=5.90e-03) 22279577 (p=7.70e-03) |
| 5 | 920 | CD4 | 1912 | 4.93E-11 | 17632570 (p=7.01e-04) 25266850 (p=2.30e-03) 21284901 (p=2.40e-03) 2990682 (p=2.80e-03) 18808677 (p=3.70e-03) 21880749 (p=4.10e-03) 27170746 (p=4.50e-03) 22693444 (p=5.90e-03) 23308067 (p=6.00e-03) 18704932 (p=6.60e-03) |
| 6 | 1803 | DPP4 | 404 | 2.42E-10 | 23831647 (p=6.98e-05) 24342026 (p=7.80e-05) 25211075 (p=8.32e-05) 23486063 (p=8.33e-05) 26701103 (p=8.54e-05) 27750111 (p=9.76e-05) 24257613 (p=2.00e-04) 26889022 (p=2.04e-04) 24067970 (p=4.00e-04) 28118607 (p=6.03e-04) |
| 7 | 3456 | IFNB1 | 376 | 2.65E-09 | 24872591 (p=6.99e-05) 24362959 (p=8.40e-05) 20181693 (p=3.05e-04) 24850742 (p=2.30e-03) 26320399 (p=2.90e-03) 28404846 (p=3.70e-03) 18708672 (p=5.90e-03) 26196448 (p=6.40e-03) 22629479 (p=9.00e-03) |
| 8 | 30835 | CD209 | 392 | 3.34E-09 | 17715238 (p=9.60e-05) 20359516 (p=1.00e-04) 18697825 (p=1.03e-04) 17041212 (p=1.04e-04) 23388721 (p=1.10e-04) 17632570 (p=7.01e-04) 24620896 (p=3.70e-03) 26018157 (p=4.70e-03) 22102941 (p=9.40e-03) |
| 9 | 3105 | HLA-A | 1379 | 4.01E-09 | 18186801 (p=7.14e-05) 18827882 (p=1.07e-04) 20844028 (p=3.04e-04) 20003377 (p=7.00e-04) 17632570 (p=7.01e-04) 28476759 (p=9.02e-04) 18808677 (p=3.70e-03) 17509453 (p=4.00e-03) 22819798 (p=5.90e-03) 25659158 (p=6.40e-03) |
| 10 | 10410 | IFITM3 | 84 | 3.78E-09 | 29263263 (p=8.05e-05) 24367104 (p=3.06e-04) 21253575 (p=6.04e-04) 27707929 (p=7.10e-03) 27492307 (p=8.10e-03) 25422070 (p=8.90e-03) |
| 11 | 1234 | CCR5 | 1424 | 4.86E-09 | 18989363 (p=1.30e-03) 15821335 (p=1.40e-03) 19124913 (p=2.80e-03) 25939314 (p=3.20e-03) 12010355 (p=3.30e-03) 18706447 (p=5.10e-03) 25918237 (p=5.40e-03) 12215252 (p=5.60e-03) 27139347 (p=5.60e-03) 18763111 (p=6.80e-03) |
| 12 | 3106 | HLA-B | 1850 | 1.02E-07 | 18186801 (p=7.14e-05) 12969506 (p=7.89e-05) 20003377 (p=7.00e-04) 17632570 (p=7.01e-04) 26070312 (p=1.50e-03) 18808677 (p=3.70e-03) 17509453 (p=4.00e-03) 19703245 (p=6.40e-03) 20374314 (p=7.70e-03) 22320938 (p=8.50e-03) |
| 13 | 9407 | TMPRSS11D | 21 | 1.07E-07 | 21994442 (p=8.49e-05) 24227843 (p=9.43e-05) 22558251 (p=9.01e-04) 23192872 (p=1.30e-03) |
| 14 | 3661 | IRF3 | 369 | 7.39E-07 | 24872591 (p=6.99e-05) 27094905 (p=7.54e-05) 17761676 (p=2.03e-04) 24622840 (p=3.70e-03) 25481026 (p=3.80e-03) 22647704 (p=5.60e-03) 15220448 (p=8.80e-03) |
| 15 | 340061 | TMEM173 | 127 | 2.21E-06 | 25212897 (p=9.70e-05) 22312431 (p=2.30e-03) 24622840 (p=3.70e-03) 25311841 (p=5.10e-03) 29263267 (p=6.60e-03) |
| 16 | 290 | ANPEP | 132 | 2.52E-06 | 11559807 (p=1.50e-03) 1350662 (p=1.70e-03) 15840518 (p=3.20e-03) 15280478 (p=7.60e-03) 12551991 (p=8.80e-03) |
| 17 | 4153 | MBL2 | 713 | 3.61E-06 | 16170752 (p=3.00e-04) 16185324 (p=3.05e-04) 15838797 (p=8.01e-04) 20712489 (p=1.90e-03) 20573835 (p=2.10e-03) 18582923 (p=3.70e-03) 16681863 (p=6.40e-03) 24601758 (p=9.50e-03) |
| 18 | 3627 | CXCL10 | 544 | 7.77E-06 | 19590927 (p=8.09e-05) 15919935 (p=6.05e-04) 16195357 (p=2.50e-03) 17052299 (p=2.60e-03) 23144331 (p=3.70e-03) 22393386 (p=7.70e-03) 16920957 (p=7.90e-03) |
| 19 | 4210 | MEFV | 345 | 9.21E-06 | 22281876 (p=3.10e-03) 24071932 (p=3.30e-03) 24433404 (p=7.80e-03) 15643295 (p=7.80e-03) 27659338 (p=9.00e-03) 24708999 (p=9.70e-03) |
| 20 | 4599 | MX1 | 179 | 9.18E-06 | 16824203 (p=6.02e-04) 20462354 (p=1.40e-03) 15135736 (p=2.90e-03) 22647704 (p=5.60e-03) 25542463 (p=9.00e-03) |
| 21 | 1514 | CTSL | 216 | 2.21E-05 | 26953343 (p=6.60e-05) 23536651 (p=6.04e-04) 27729455 (p=7.01e-04) 18971274 (p=9.02e-04) 26374357 (p=7.60e-03) |
| 22 | 3123 | HLA-DRB1 | 2531 | 4.24E-05 | 18186801 (p=7.14e-05) 18827882 (p=1.07e-04) 20003377 (p=7.00e-04) 20193583 (p=9.00e-04) 19445991 (p=3.30e-03) 17509453 (p=4.00e-03) 15462607 (p=4.50e-03) 15256088 (p=6.30e-03) 19703245 (p=6.40e-03) 17257320 (p=8.10e-03) |
| 23 | 10747 | MASP2 | 112 | 6.00E-05 | 19405982 (p=1.11e-04) 19737459 (p=8.01e-04) 18582923 (p=3.70e-03) 27725284 (p=8.90e-03) |
| 24 | 2875 | GPT | 123 | 8.35E-05 | 15306699 (p=3.01e-04) 23880909 (p=3.30e-03) 22975640 (p=4.50e-03) 27304617 (p=8.90e-03) |
| 25 | 3107 | HLA-C | 896 | 1.44E-04 | 21958371 (p=1.10e-04) 17632570 (p=7.01e-04) 18808677 (p=3.70e-03) 17509453 (p=4.00e-03) 16455884 (p=5.60e-03) 22474021 (p=6.80e-03) 18987136 (p=8.80e-03) |
| 26 | 684 | BST2 | 333 | 1.48E-04 | 22647704 (p=5.60e-03) 23308067 (p=6.00e-03) 22509177 (p=6.00e-03) 24465210 (p=8.90e-03) 22171785 (p=9.00e-03) |
| 27 | 91937 | TIMD4 | 42 | 1.96E-04 | 23555248 (p=3.02e-04) 27122575 (p=2.90e-03) 17407086 (p=7.20e-03) |
| 28 | 7124 | TNF | 5360 | 3.79E-04 | 17223386 (p=9.01e-04) 25081719 (p=9.01e-04) 18312678 (p=2.30e-03) 22276993 (p=2.80e-03) 17532082 (p=2.80e-03) 20041225 (p=3.40e-03) 17666321 (p=3.80e-03) 26939244 (p=5.10e-03) 18708672 (p=5.90e-03) 20732366 (p=7.60e-03) |
| 29 | 55096 | EBLN2 | 6 | 6.04E-04 | 20054395 (p=4.50e-03) 20686665 (p=6.90e-03) |
| 30 | 340900 | EBLN1 | 6 | 5.84E-04 | 20054395 (p=4.50e-03) 20686665 (p=6.90e-03) |
| 31 | 8519 | IFITM1 | 65 | 6.44E-04 | 29263263 (p=8.05e-05) 21253575 (p=6.04e-04) 25422070 (p=8.90e-03) |
| 32 | 114548 | NLRP3 | 484 | 7.23E-04 | 27101784 (p=5.60e-03) 19826485 (p=8.50e-03) 24501247 (p=9.10e-03) 21058222 (p=9.50e-03) 24708999 (p=9.70e-03) |
| 33 | 29110 | TBK1 | 247 | 9.56E-04 | 24872591 (p=6.99e-05) 21364999 (p=9.41e-05) 24622840 (p=3.70e-03) 24549848 (p=8.60e-03) |
| 34 | 950 | SCARB2 | 86 | 1.37E-03 | 23959904 (p=2.03e-04) 19543282 (p=9.01e-04) 22272359 (p=4.50e-03) |
| 35 | 6440 | SFTPC | 102 | 2.21E-03 | 19148933 (p=7.00e-04) 20403820 (p=3.40e-03) 20656946 (p=8.90e-03) |
| 36 | 7706 | TRIM25 | 110 | 2.69E-03 | 28148787 (p=8.02e-05) 27122586 (p=4.00e-03) 26138103 (p=6.50e-03) |
| 37 | 119710 | C11orf74 | 16 | 3.78E-03 | 18433331 (p=1.50e-03) 16157265 (p=5.40e-03) |
| 38 | 2524 | FUT2 | 127 | 3.92E-03 | 22025362 (p=5.90e-03) 25037042 (p=8.20e-03) 12692541 (p=9.50e-03) |
| 39 | 339390 | CLEC4G | 18 | 4.57E-03 | 18697825 (p=1.03e-04) 19111020 (p=3.30e-03) |
| 40 | 5478 | PPIA | 395 | 4.86E-03 | 26318518 (p=9.40e-05) 25445708 (p=3.40e-03) 15688292 (p=5.10e-03) 25556234 (p=7.20e-03) |
| 41 | 3458 | IFNG | 1755 | 5.42E-03 | 24475220 (p=3.10e-03) 18708672 (p=5.90e-03) 20732366 (p=7.60e-03) 15643599 (p=7.90e-03) 12590978 (p=8.90e-03) 18937577 (p=9.50e-03) 21533215 (p=9.50e-03) |
| 42 | 4864 | NPC1 | 148 | 5.59E-03 | 21866101 (p=1.50e-03) 21866103 (p=2.80e-03) 22395071 (p=6.90e-03) |
| 43 | 6772 | STAT1 | 803 | 5.73E-03 | 19656875 (p=8.01e-04) 17596301 (p=8.01e-04) 26719274 (p=4.10e-03) 22647704 (p=5.60e-03) 21533215 (p=9.50e-03) |
| 44 | 3669 | ISG20 | 22 | 6.11E-03 | 21036379 (p=4.50e-03) 22647704 (p=5.60e-03) |
| 45 | 9636 | ISG15 | 162 | 6.81E-03 | 28931677 (p=6.35e-05) 17692280 (p=5.60e-03) 22647704 (p=5.60e-03) |
| 46 | 6556 | SLC11A1 | 178 | 8.81E-03 | 17223386 (p=9.01e-04) 17067929 (p=4.10e-03) 12447767 (p=8.90e-03) |
| 47 | 3119 | HLA-DQB1 | 1404 | 8.97E-03 | 23710940 (p=7.00e-04) 15462607 (p=4.50e-03) 25200477 (p=8.90e-03) 25197808 (p=9.20e-03) 28359736 (p=9.40e-03) 14988284 (p=9.70e-03) |
| 48 | 282617 | IFNL3 | 490 | 9.20E-03 | 23950709 (p=3.70e-03) 27155288 (p=5.30e-03) 21861668 (p=8.10e-03) 22168813 (p=8.50e-03) |
| 49 | 81844 | TRIM56 | 29 | 9.62E-03 | 25253338 (p=8.02e-04) 26889027 (p=9.02e-04) |
| 50 |  | BRD2/4 | - | Manual Curation | https://doi.org/10.1101/2020.03.22.002386 |
| 51 | 1459 | CSNK2A2 | - | Manual Curation | https://doi.org/10.1101/2020.03.22.002386 |
| 52 | 3066 | HDAC2 | - | Manual Curation | https://doi.org/10.1101/2020.03.22.002386 |
| 53 | 537 | ATP6AP1 | - | Manual Curation | https://doi.org/10.1101/2020.03.22.002386 |
| 54 | 523 | ATP6V1A | - | Manual Curation | https://doi.org/10.1101/2020.03.22.002386 |
| 55 | 10280 | SIGMAR1 | - | Manual Curation | https://doi.org/10.1101/2020.03.22.002386 |
| 56 | 27346 | TMEM97 | - | Manual Curation | https://doi.org/10.1101/2020.03.22.002386 |
| 57 | 1312 | COMT | - | Manual Curation | https://doi.org/10.1101/2020.03.22.002386 |
| 58 | 80142 | PTGES2 | - | Manual Curation | https://doi.org/10.1101/2020.03.22.002386 |
| 59 | 4719 | NDUFS1 | - | Manual Curation | https://doi.org/10.1101/2020.03.22.002386 |
| 60 | 8737 | RIPK1 | - | Manual Curation | https://doi.org/10.1101/2020.03.22.002386 |
| 61 | 5566 | PRKACA | - | Manual Curation | https://doi.org/10.1101/2020.03.22.002386 |
| 62 | 3615 | IMPDH2 | - | Manual Curation | https://doi.org/10.1101/2020.03.22.002386 |
| 63 | 2717 | GLA | - | Manual Curation | https://doi.org/10.1101/2020.03.22.002386 |
| 64 | 1786 | DNMT1 | - | Manual Curation | https://doi.org/10.1101/2020.03.22.002386 |
| 65 | 4015 | LOX | - | Manual Curation | https://doi.org/10.1101/2020.03.22.002386 |
| 66 | 2011 | MARK2 | - | Manual Curation | https://doi.org/10.1101/2020.03.22.002386 |
| 67 | 79077 | DCTPP1 | - | Manual Curation | https://doi.org/10.1101/2020.03.22.002386 |
| 68 | 4140 | MARK3 | - | Manual Curation | https://doi.org/10.1101/2020.03.22.002386 |
| 69 | 29110 | TBK1 | - | Manual Curation | https://doi.org/10.1101/2020.03.22.002386 |
| 70 | 2150 | F2RL1 | - | Manual Curation | https://doi.org/10.1101/2020.03.22.002386 |
| 71 | 4363 | ABCC1 | - | Manual Curation | https://doi.org/10.1101/2020.03.22.002386 |
| 72 | 23367 | LARP1 | - | Manual Curation | https://doi.org/10.1101/2020.03.22.002386 |
| 73 | 23307 | FKBP15 | - | Manual Curation | https://doi.org/10.1101/2020.03.22.002386 |
| 74 | 51661 | FKBP7 | - | Manual Curation | https://doi.org/10.1101/2020.03.22.002386 |
| 75 | 9470 | EIF4E2 | - | Manual Curation | https://doi.org/10.1101/2020.03.22.002386 |
| 76 |  | NUPs | - | Manual Curation | https://doi.org/10.1101/2020.03.22.002386 |
| 77 | 8480 | RAE1 | - | Manual Curation | https://doi.org/10.1101/2020.03.22.002386 |
| 78 | 91754 | NEK9 | - | Manual Curation | https://doi.org/10.1101/2020.03.22.002386 |
| 79 | 11190 | CEP250 | - | Manual Curation | https://doi.org/10.1101/2020.03.22.002386 |
| 80 | 51661 | FKBP7 | - | Manual Curation | https://doi.org/10.1101/2020.03.22.002386 |
| 81 | 60681 | FKBP10 | - | Manual Curation | https://doi.org/10.1101/2020.03.22.002386 |
| 82 | 8453 | CUL2 | - | Manual Curation | https://doi.org/10.1101/2020.03.22.002386 |

**Table S4: The top 10 enriched pathways miRNA-TF-gene coregulatory network that was constructed using 82 β-coronavirus genes curated from the literature**

| **Pathway identifier** | **Pathway name** | **Entities pValue** | **\|log(p.Value)** | **Entities FDR** | **#Reactions found** | **Species identifier** |
| --- | --- | --- | --- | --- | --- | --- |
| R-HSA-1236977 | Endosomal/Vacuolar pathway | 1.11E-16 | 53.00029 | 5.88E-15 | 4 | 9606 |
| R-HSA-877300 | Interferon gamma signaling | 1.11E-16 | 53.00029 | 5.88E-15 | 15 | 9606 |
| R-HSA-909733 | Interferon alpha/beta signaling | 1.11E-16 | 53.00029 | 5.88E-15 | 18 | 9606 |
| R-HSA-983170 | Antigen Presentation: Folding, assembly and peptide loading of class I MHC | 1.11E-16 | 53.00029 | 5.88E-15 | 13 | 9606 |
| R-HSA-913531 | Interferon Signaling | 1.11E-16 | 53.00029 | 5.88E-15 | 50 | 9606 |
| R-HSA-983169 | Class I MHC mediated antigen processing & presentation | 1.11E-16 | 53.00029 | 5.88E-15 | 20 | 9606 |
| R-HSA-1280218 | Adaptive Immune System | 1.11E-16 | 53.00029 | 5.88E-15 | 80 | 9606 |
| R-HSA-1236975 | Antigen processing-Cross presentation | 1.11E-16 | 53.00029 | 5.88E-15 | 7 | 9606 |
| R-HSA-1236974 | ER-Phagosome pathway | 1.11E-16 | 53.00029 | 5.88E-15 | 3 | 9606 |

.

**Table S5: Z-scores of different subgraphs calculated after randomization of miRNA-TF-gene-coregulatory network.**

| Motif diagrammatic represeentation | Subgraph ID | Z-score |
| --- | --- | --- |
| 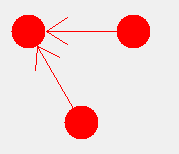 | 000100100 | -0.995786541479132 |
| 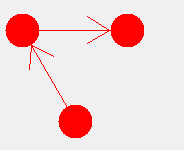 | 010000100 | -0.9992400260230015 |
| 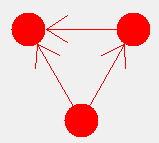 | 000100110 | -0.907322222017 |
| 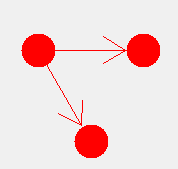 | 011000000 | -0.999976311663009967 |

**Table S6: The list of the genes expressed or regulated during High altitude pulmonary edema.**

| **S.NO** | **HAPE Genes** | **PMID** |
| --- | --- | --- |
| 1 | ACE | 17071838 |
| 2 | NOS3 | 17099031 |
| 3 | AGT | 21393362 |
| 4 | AGTR1 | 27732943 |
| 5 | IL1RN | 30672138 |
| 6 | GSTP1 | 1158083 |
| 7 | GSTM1 | 1158083 |
| 8 | CYBA | 21973220 |
| 9 | GSTT1 | 21190504 |
| 10 | ACYP2 | 28353602 |
| 11 | IL1A | 30672138 |
| 12 | PON1 | 24067188 |
| 13 | IL10 | 9628235 |
| 14 | IL1B | 29247995 |
| 15 | TNF | 21188088 |
| 16 | IL6 | 29247995 |
| 17 | APOE | 29247995 |
| 18 | ET-1 | 26984355 |
| 19 | sKDR | 26984355 |
| 20 | Angptl4 | 26984355 |
| 21 | ST1A1 | 31444657 |
| 22 | ACADM | 24465776 |
| 23 | ADH1A | 24465776 |
| 24 | ADH1B | 24465776 |
| 25 | ADH1C | 24465776 |
| 26 | ADRA1D | 24465776 |
| 27 | ARAF | 24465776 |
| 28 | ARHGEF11 | 24465776 |
| 29 | ARRB2 | 24465776 |
| 30 | ATP6V1C1 | 24465776 |
| 31 | ATP6V1F | 24465776 |
| 32 | CCL24 | 24465776 |
| 33 | CCR8 | 24465776 |
| 34 | CLDN10 | 24465776 |
| 35 | CLDN23 | 24465776 |
| 36 | CLDN6 | 24465776 |
| 37 | COX1 | 24465776 |
| 38 | COX2 | 24465776 |
| 39 | CPT2 | 24465776 |
| 40 | CXCL16 | 24465776 |
| 41 | CYP4A11 | 24465776 |
| 42 | DRD1 | 24465776 |
| 43 | EDNRA | 24465776 |
| 44 | FGF7 | 24465776 |
| 45 | GLG1 | 24465776 |
| 46 | GNB2 | 24465776 |
| 47 | GRM5 | 24465776 |
| 48 | HADHA | 24465776 |
| 49 | JAK2 | 24465776 |
| 50 | KCNMA1 | 24465776 |
| 51 | KDELR3 | 24465776 |
| 52 | MADCAM1 | 24465776 |
| 53 | MAP2K2 | 24465776 |
| 54 | MAP2K5 | 24465776 |
| 55 | MAP2K7 | 24465776 |
| 56 | MAPK10 | 24465776 |
| 57 | MT-CO3 | 24465776 |
| 58 | MT-ND1 | 24465776 |
| 59 | MT-ND2 | 24465776 |
| 60 | MT-ND4 | 24465776 |
| 61 | MT-ND5 | 24465776 |
| 62 | MT-ND6 | 24465776 |
| 63 | MUC2 | 24465776 |
| 64 | MYL2 | 24465776 |
| 65 | MYLK | 24465776 |
| 66 | MYLK3 | 24465776 |
| 67 | NCKAP1L | 24465776 |
| 68 | NDUFA10 | 24465776 |
| 69 | NDUFAB1 | 24465776 |
| 70 | NDUFS1 | 24465776 |
| 71 | NDUFV1 | 24465776 |
| 72 | NFATC1 | 24465776 |
| 73 | NFATC3 | 24465776 |
| 74 | NLGN3 | 24465776 |
| 75 | NOX1 | 24465776 |
| 76 | NRXN3 | 24465776 |
| 77 | PAK3 | 24465776 |
| 78 | PLA2G1B | 24465776 |
| 79 | PLCB1 | 24465776 |
| 80 | PPARA | 24465776 |
| 81 | PRKAG2 | 24465776 |
| 82 | PTK2 | 24465776 |
| 83 | PXN | 24465776 |
| 84 | RASGRP2 | 24465776 |
| 85 | RELA | 24465776 |
| 86 | SEC61A1 | 24465776 |
| 87 | SEC61A2 | 24465776 |
| 88 | SH2D2A | 24465776 |
| 89 | SOS1 | 24465776 |
| 90 | TUBA1B | 24465776 |
| 91 | UQCRFS1 | 24465776 |
| 92 | VCAM1 | 24465776 |
| 93 | UQCRB | 24465776 |

**Table S7: The list of the 15 FFLs present in miRNA-TF-gene coregulatory network that are having common HAPE TFs/genes.**

| **TF** | **Gene** | **miRNA** | **miRNAs probable role in COVID-19** | **miRNA role in lung disorder** |
| --- | --- | --- | --- | --- |
| PPARA | SOD2 | hsa-mir-9-5p | ACE2 receptor dysregulation | TGF-Beta signalling |
| REL | IL10 | hsa-mir-98-5p | Antiviral host miRNA against COVID-19 | TGF-Beta signalling |
| POU2F1 | VCAM1 | hsa-mir-1277-5p | Novel | Novel |
| CEBPD | IL6 | hsa-mir-98-5p | Antiviral host miRNA against COVID-19 | TGF-Beta signalling |
| MYC | MAP2K7 | hsa-mir-149-3p | Not known | Lung carcinoma |
| REST | IL1RN | hsa-mir-125a-5p | Antiviral host miRNA against COVID-19 | Lung carcinoma |
| PPARA | IL6 | hsa-mir-9-5p | ACE2 receptor dysregulation | TGF-Beta signalling |
| EP300 | VCAM1 | hsa-mir-23b-3p | Antiviral host miRNA against COVID-19 | TGF_Beta signalling |
| HOXA9 | VCAM1 | hsa-mir-1277-5p | Novel | Novel |
| EP300 | IL6 | hsa-mir-149-5p | Novel | Lung carcinoma |
| FOXO1 | IL6 | hsa-mir-9-5p | ACE2 receptor dysregulation | TGF-Beta signalling |
| FOXO1 | IL6 | hsa-mir-98-5p | Antiviral host miRNA against COVID-19 | TGF-Beta signalling |
| PPARA | IL6 | hsa-mir-9-5p | ACE2 receptor dysregulation | TGF-Beta signalling |
| REL | IL6 | hsa-mir-365a-3p | Novel | Lung carcinoma |
| REL | IL6 | hsa-mir-98-5p | Antiviral host miRNA against COVID-19 | TGF-Beta signalling |

**Table S8: The list of TFs regulating SERPINE1 in miRNA-TF-gene coregulatory network.**

| TF | TF_entrez | Gene | Gene entrez | PMID |
| --- | --- | --- | --- | --- |
| ARNTL | 406 | SERPINE1 | 5054 | 10894149 |
| ARNTL2 | 56938 | SERPINE1 | 5054 | 22846578 |
| CEBPA | 1050 | SERPINE1 | 5054 | 19767562 |
| CEBPD | 1052 | SERPINE1 | 5054 | 17350599 |
| E2F1 | 1869 | SERPINE1 | 5054 | 17498959 |
| EP300 | 2033 | SERPINE1 | 5054 | 22955619 |
| ESR1 | 2099 | SERPINE1 | 5054 | 17047041 |
| ESR2 | 2100 | SERPINE1 | 5054 | 17498959 |
| FOS | 2353 | SERPINE1 | 5054 | 22955619 |
| FOXA1 | 3169 | SERPINE1 | 5054 | 22955619 |
| JUN | 3725 | SERPINE1 | 5054 | 22955619 |
| KLF10 | 7071 | SERPINE1 | 5054 | 10506168 |
| MYC | 4609 | SERPINE1 | 5054 | 22955619 |
| NFIC | 4782 | SERPINE1 | 5054 | 20966046 |
| NFKB1 | 4790 | SERPINE1 | 5054 | 15317818 |
| NR3C1 | 2908 | SERPINE1 | 5054 | 22955619 |
| PARP1 | 142 | SERPINE1 | 5054 | 15781633 |
| PPARA | 5465 | SERPINE1 | 5054 | 17533737 |
| PPARG | 5468 | SERPINE1 | 5054 | 17533737 |
| RXRA | 6256 | SERPINE1 | 5054 | 22955619 |
| SMAD3 | 4088 | SERPINE1 | 5054 | 10506168 |
| SMAD4 | 4089 | SERPINE1 | 5054 | 19853299 |
| SP1 | 6667 | SERPINE1 | 5054 | 10029407 |
| SRF | 6722 | SERPINE1 | 5054 | 19853299 |
| TGIF1 | 7050 | SERPINE1 | 5054 | 20646316 |
| USF1 | 7391 | SERPINE1 | 5054 | 20646316 |
